# Nearly Complete Genome Sequence of a Newcastle Disease Virus Strain Isolated from a Wild Garganey (*Spatula querquedula*) in Russia

**DOI:** 10.1128/MRA.01072-19

**Published:** 2019-12-12

**Authors:** Alexandra V. Glushchenko, Tatyana Y. Alikina, Kseniya S. Yurchenko, Egor V. Shekunov, Marina A. Gulyaeva, Keita Matsuno, Masatoshi Okamatsu, Marsel R. Kabilov, Alexander M. Shestopalov

**Affiliations:** a Federal Research Center of Fundamental and Translational Medicine, Research Institute of Experimental and Clinical Medicine, Novosibirsk, Russia; b Institute of Chemical Biology and Fundamental Medicine, Siberian Branch of the Russian Academy of Sciences, Novosibirsk, Russia; c Novosibirsk State University, Novosibirsk, Russia; d Hokkaido University, Sapporo, Japan; Portland State University

## Abstract

This work describes the nearly complete genome sequence of Newcastle disease virus (NDV) strain NDV/Novosibirsk/garganey/27/2014, which was isolated from a wild garganey in western Siberia, Russia. The NDV strain was classified as belonging to class II of genotype I and was identified as having recent common ancestry with isolates from wild and domestic birds in China and South Korea.

## ANNOUNCEMENT

Virulent strains of Newcastle disease virus (NDV) cause economically significant disease in wild birds and poultry (1). NDV isolates from apparently healthy waterfowl cause mild infections in wild birds of more than 250 species (2). NDV is an enveloped, negative-sense, single-stranded, nonsegmented RNA virus belonging to the *Paramyxoviridae* family (3). NDV strains have variable pathogenicity and are divided into three groups, depending on the severity of disease in chickens, i.e., velogenic, mesogenic, or lentogenic (4).

In the present study, we report the nearly complete genome sequence of NDV strain NDV/Novosibirsk/garganey/27/2014, which was detected in conventional cloacal swabs from a wild garganey (Spatula querquedula) in 2014 in western Siberia, Russia. The sample, obtained by virus isolation from 9-day-old chicken embryos according to standard procedures, was found to contain NDV (5). The carrier bird had no clinical signs or symptoms of Newcastle disease. Standard pathogenicity assays were performed, including determination of the mean death time (MDT) and the intracerebral pathogenicity index (ICPI) (5). The pathogenicity indexes (MDT of 124 h and ICPI of 0.4) indicated that NDV/Novosibirsk/garganey/27/2014 is lentogenic.

RNA was isolated from 100 μl of chicken allantoic fluid with cultured viral particles using a GeneJET viral DNA/RNA purification kit (Thermo Fisher Scientific) and was treated with Turbo DNase (Thermo Fisher Scientific). Up to 40 ng of RNA was used for the DNA library, which was prepared by using the TruSeq RNA sample preparation kit version 2 (random hexamers are used for reverse transcription), without using oligo(dT) beads (Illumina). The DNA libraries were sequenced with reagent kit version 3 (600-cycle format) on a MiSeq genome sequencer (Illumina) at the Siberian Branch of the Russian Academy of Sciences Genomics Core Facility (Novosibirsk, Russia). The complete coding genome was assembled *de novo* with CLC Genomics Workbench version 8.5 (Qiagen), with default parameters. After trimming (*q* values of >20) and removal of adapter sequences, about 3,000 reads were assembled into a genome of 15,169 nucleotides at 37-fold coverage, with a GC content of 47.4%.

The amino acid sequence of the proteolytic F protein cleavage site, as the main predictor of virulence, contains 112G-KQG-R116 with leucine at residue 117, indicating that NDV/Novosibirsk/garganey/27/2014 is an avirulent strain. Strain NDV/Novosibirsk/garganey/27/2014 was phylogenetically compared with other strains based on the F gene coding sequences. It was shown that the F gene from NDV/Novosibirsk/garganey/27/2014 is similar to that of the NDV isolate KR/duck/13/07 (DK13), which belongs to class II of genotype I in terms of the genome analysis (6). The results of a BLASTn (version 2.10.0+) search of the nucleotide database revealed that NDV/Novosibirsk/garganey/27/2014 has a structure most homologous to the complete coding genome sequence of NDV isolate DK13 from South Korea (GenBank accession no. KT186351; length, 15,186 bp) and NDV isolate R8 from China (HM063424; length, 15,186 bp), with 97.89% and 97.73% nucleotide identities, respectively, and various NDV strains isolated from Anser fabalis in China in 2016, with 97.68% (MH289840; length, 15,186 bp) and 97.67% (MH289837; length, 15,184 kb) nucleotide identities. The listed strains were isolated from poultry (domestic duck) and wild migratory birds (water rail and bean goose); this finding suggests the ability of carrier birds without clinical signs of disease to be responsible for transmitting NDV to both wild and domesticated birds (7, 8).

### Data availability.

The nearly complete genome sequence of and associated data for NDV/Novosibirsk/garganey/27/2014 have been deposited in GenBank under accession no. KU662357. Raw sequencing data are registered in the NCBI SRA database under accession no. SRP221531.
